# Frequency-Specific Transcranial Photobiomodulation Elicits Complementary Glial Mechanisms for Neurovascular Protection and Amyloid Clearance in Alzheimer Disease

**DOI:** 10.34133/cbsystems.0551

**Published:** 2026-06-23

**Authors:** Bowen Zhang, Zemeng Chen, Weiguang Li, Louzhe Xu, Songqi Yang, Felix Wang, Kai Yan, Xunbin Wei, Ting Li

**Affiliations:** ^1^ Biomedical Engineering Institute, Chinese Academy of Medical Sciences and Peking Union Medical College, Tianjin 300192, China.; ^2^ Department of Psychology, The State Key Laboratory of Brain and Cognitive Sciences, The University of Hong Kong, Hong Kong SAR 999077, China.; ^3^ Department of Biosystems, KU Leuven, Leuven 3001, Belgium.; ^4^ Children Hospital of Fudan University, Shanghai 201102, China.; ^5^ Department of Biomedical Engineering, College of Future Technology, Peking University, Beijing 100871, China.; ^6^ Institute of Intelligent Medicine, Chinese Academy of Medical Sciences and Peking Union Medical College, Beijing 100730, China.

## Abstract

Alzheimer disease (AD), a devastating neurodegenerative disorder, is pathologically defined by amyloid-β (Aβ) deposition and neurofibrillary tangles. Critically, concomitant cerebrovascular dysfunction compromises neuronal homeostasis and significantly accelerates AD progression by impairing the neurovascular unit. However, effective strategies to modulate this complex neurovascular pathology remain unclear. Here, we applied transcranial photobiomodulation (tPBM) with continuous-wave (CW) and 40-Hz pulsed light to target neurovascular pathology in 5xFAD mice. The results showed that both tPBM modalities comparably ameliorated cognitive dysfunction through distinct glial-mediated mechanisms. Specifically, CW light primarily enhanced astrocyte–vascular coupling, which ameliorated vascular dysfunction and protected synapses. In contrast, 40-Hz light predominantly drove spatial redistribution of microglia toward Aβ plaques, thereby enhancing localized amyloid clearance. These findings reveal complementary pathways for tPBM in AD intervention, highlighting that CW and 40-Hz light offer modality-specific therapeutic advantages: The former targets cerebrovascular dysfunction, while the latter addresses Aβ plaque deposition. Collectively, our study provides critical mechanistic insights for optimizing tPBM protocols, establishing a foundation for more precise and comprehensive AD interventions.

## Introduction

Alzheimer disease (AD), as the most common form of dementia worldwide, was mainly characterized by abnormal deposition of β-amyloid (Aβ) plaques and neuronal degeneration [1–3]. Numerous studies have confirmed that AD was also usually accompanied by cerebral vascular pathology, including the reduction and distortion of small blood vessels and neurovascular unit (NVU) dysfunction [4–6]. While recent Aβ-targeting therapies show promise in early-stage AD [7,8], they fail to ameliorate overall neurodegenerative pathology and concomitant vascular impairments. Moreover, anti-Aβ therapies exhibited strong dose dependency and had the risk of amyloid-related imaging abnormalities, including microhemorrhages and cerebral edema [9,10]. Therefore, developing novel sustainable therapeutic approaches to complement existing anti-Aβ therapies holds significant value for effective AD intervention.

Photobiomodulation (PBM) has emerged in recent years as a promising noninvasive light therapy for neurological diseases with fewer side effects [11–13]. Transcranial PBM (tPBM) with continuous-wave (CW) light has been shown to mitigate brain degeneration and counteract neuroinflammation in AD mice. These effects likely involve complex cellular interactions, particularly the potential activation of glial cells [14,15]. Moreover, CW near-infrared (NIR) light also modulates vascular-endothelial-growth-factor-mediated angiogenesis [16] and regulates nitric oxide (NO) release to improve hemodynamics [17,18]. In contrast, pulsed-wave (PW) light therapy protocols for AD mice primarily focus on sensory stimulation to induce nerve entrainment [16,19–22]. Numerous research highlights that 40-Hz visual flickering stimulation attenuates AD pathology by gamma-frequency entrainment [19,22,23]. These activation subsequently reduces Aβ production and enhances microglia-mediated endocytosis [23,24]. While these sensory approaches are promising, the specific potential of direct transcranial PW light stimulation in AD remains largely unexplored. Given evidence from traumatic brain injury (TBI) and stroke models indicating that pulsed light may yield superior neuroprotective outcomes compared to CW light [25–27], a comparative evaluation of transcranial CW versus PW light efficacy and mechanisms is urgently needed to optimize tPBM strategies for AD.

The neurovascular impairment in AD is tightly interwoven with glial dysfunction, specifically astrocytes and microglia [28–31]. As the most abundant glial cells in the brain, astrocytes not only regulate synaptic plasticity but also serve as essential components of both the blood–brain barrier and NVU [32,33]. The astrocytic atrophy has been found in the early stage of AD [34,35]. Smaller cell bodies and less complex processes of astrocytes in AD diminished the synaptic maintenance, thereby contributing to synaptic loss and neurotransmission impairment [36,37]. The degeneration of the end-feet structure of astrocytes affected the function of the blood–brain barrier and the regulation of cerebral blood flow [28,29]. Microglia were mainly activated in AD to phagocytose and degrade Aβ aggregates, limiting plaque formation [24,38]. Microglia also played an important role in vascular protection, mainly manifested in regulating cerebral blood flow [39] and decreasing cerebral amyloid angiopathy [31,40]. Notably, over-activated microglia phagocytose synapses, leading to synaptic loss and also exacerbating neuroinflammation in the late stage of AD [41]. Some evidence suggests that tPBM can alleviate vascular impairment in AD models [16,24], but whether and how glial cells contribute to this protective effect remain unclear.

In this study, we explored the glial mechanisms of CW and 40-Hz light on neurovascular system in 5xFAD mice. The results revealed that both tPBM modalities improved cognitive function, while they operated through distinct target mechanisms. CW light primarily enhanced astrocyte–vascular coupling to protect the cerebrovasculature, which further supported synaptic and neuronal repair. In contrast, 40-Hz light achieved efficient Aβ clearance and synaptic protection by promoting spatial redistribution of microglia. These findings indicated the pivotal role of glial cell activation in tPBM-mediated cerebrovascular and synaptic protection and provided a foundation for optimized tPBM strategies.

## Materials and Methods

### Animals and experimental design

**Fig. 1.**
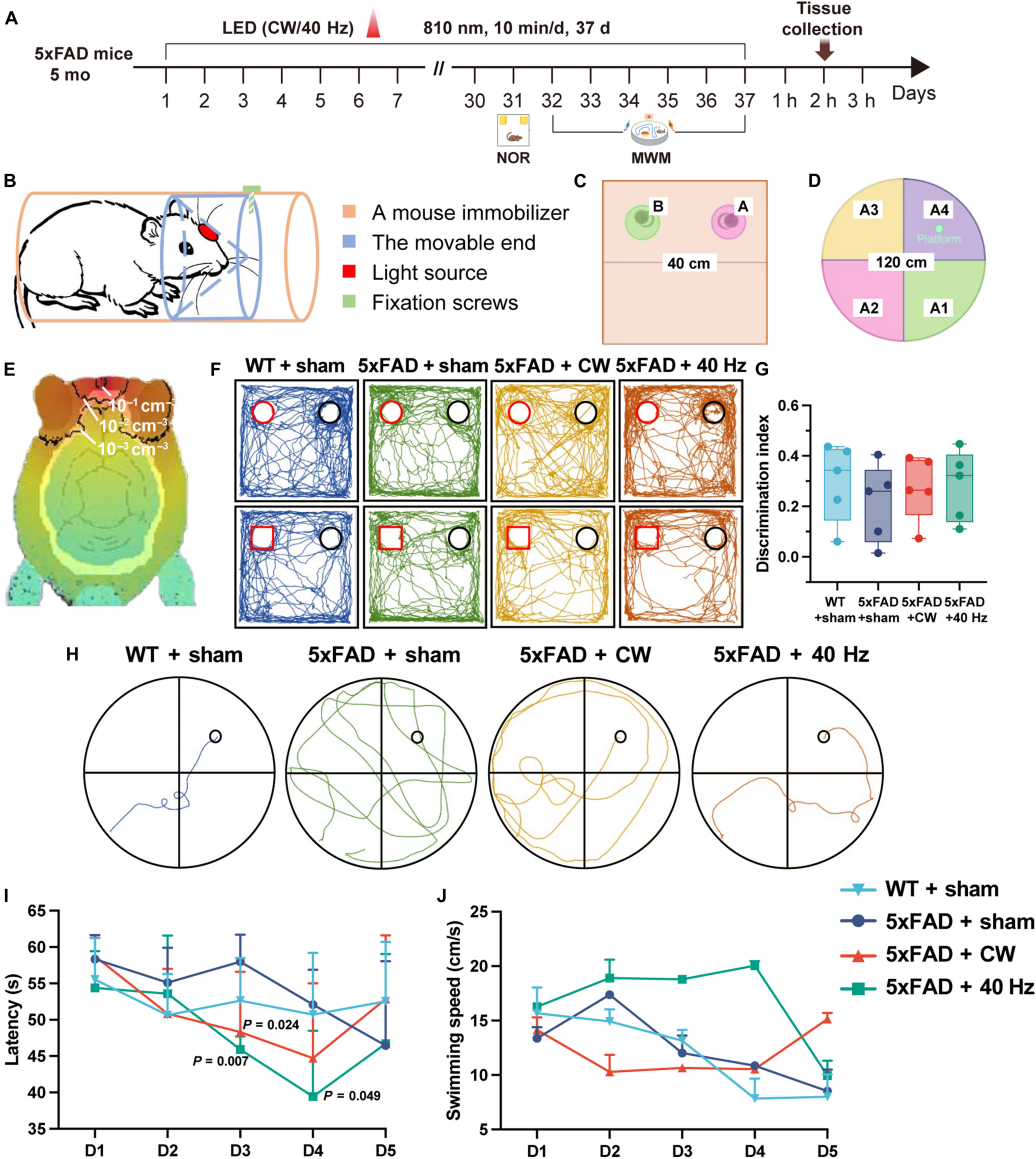
Effect of near-infrared light on cognitive dysfunction in 5xFAD mice. (A) Scheme of the experimental design. CW, continuous wave. (B) Schematic representation of a mouse undergoing transcranial photobiomodulation. (C and D) Schematic diagram of the novel object recognition (NOR) (C) and Morris water maze (MWM) (D) experimental site. (E) Photon absorption distribution from the Monte Carlo simulation. (F) Representative path of mice in NOR during training (upper) and testing (lower). WT, wild type. (G) The discrimination index during the NOR test. (H) Representative swimming path of mice on day 4. (I and J) The latency (I) and swimming speed (J) in the 4 groups during the 5-d MWM test. Data in (G) and (I and J) are analyzed by 1-way and 2-way analysis of variance (ANOVA) with Tukey post hoc tests of multiple groups, respectively.

Adult (5-mo-old) male and female 5xFAD mice with an average body weight of 24 to 28 g and age-matched littermate wild-type (WT) C57BL/6J mice were utilized in this study [42]. All mice were in the C57BL/6J background and obtained from Yangzhou Youdu Biotechnology Co. Ltd (Yangzhou, China). WT mice all received sham light treatment as controls (WT + sham, *n* = 5). All 5xFAD mice were randomly divided into the following three groups: (a) 5xFAD mice with sham NIR light (5xFAD + sham, *n* = 5); (b) 5xFAD mice with CW light (5xFAD + CW, *n* = 5); and (c) 5xFAD mice with 40-Hz light (5xFAD + 40 Hz, *n* = 5). All mice were housed at 23 ± 3 °C with constant humidity (approximately 40% to 70%) under a 12-h light/dark cycle (light phase: 8:00 AM to 8:00 PM). Up to 5 mice of the same sex and group were housed per cage with ad libitum access to standard water and food. All interventions in this study complied with the Guide for the Care and Use of Laboratory Animals. The experiment was reviewed and approved by the Institutional Animal Care and Use Committee of the Chinese Academy of Medical Sciences (approval number: IRM/1-IACUC-240510-01). Our tPBM lasted for 10 min per day and 37 consecutive days (Fig. 1A). In the last 7 d of tPBM, we sequentially employed 1-d novel object recognition (NOR) tests and 6-d Morris water maze (MWM) tests to evaluate spatial memory and learning ability in mice. After these cognitive function tests, mice were humanely euthanized, and brain tissues were collected for subsequent pathological analysis.

### Transcranial photobiomodulation with NIR light

The tPBM device comprised a control circuit and several light-emitting diode (LED) light sources (2835FIRC-81L14I100) connected in series. To ensure optimal photon transmission, the hair on the heads in mice was gently shaved using depilatory cream prior to tPBM, allowing for direct contact between the light source and the scalp (Fig. 1B). Mice were restrained in the immobilizer featuring an adjustable conical head restraint. The LED source was affixed internally within the immobilizer. The LEDs emitted light at a wavelength of 810 ± 50 nm, and the optical power density at the scalp was maintained at 25 mW/cm^2^ for both CW and 40-Hz (duty cycle 50%) groups. The spot size had a radius of approximately 0.47 cm. These parameters were selected based on previously published and widely used tPBM protocols [16,43,44], which reported effective brain penetration of 810-nm light, with approximately 25% transmission through a hairless scalp [43]. Thus, the estimated power density reaching the cerebral tissue was approximately 6.25 mW/cm^2^ for the CW group and 3.125 mW/cm^2^ for the 40-Hz group. The control group underwent identical handling procedures but remained without NIR-light exposure.

### Visualization of NIR photon distribution in the mouse brain

We employed a voxelized anatomical mouse model in conjunction with the Monte Carlo modeling of light propagation in voxelized media (MCVM) to compute and visualize the 3-dimensional distribution of 810-nm light within the realistic mouse brain. MCVM has been rigorously tested and validated through comparisons with experimental data [45,46] and gold-standard computational methods [47], making it a widely adopted tool in the biomedical optics community [48–51]. The mouse brain model was reconstructed from well-established in vivo MRI data of adult male wild-type C57BL/6J mice [52] (spatial resolution: 100 μm × 100 μm × 200 μm) and segmented into 8 distinct regions, including scalp, skull, cerebrospinal fluid, cerebral parenchyma, and so on. The optical properties of each tissue at 810 nm are shown in Table S1. We combined MCVM and voxelized structured model in computing light propagation following the study on MCVM and digital human phantom [53]. A Gaussian beam (0.2 cm diameter) was positioned at the bregma to model the tPBM, with a sufficient number of photon packets launched into the mouse brain in the MCVM computation setup. The photon absorption and scattering events of every photon packet in the voxelized mouse were tracked, statistically summarized, registered, and visualized in the voxelized 3-dimensional mouse anatomical structure. Finally, the total photon absorption in the brain parenchyma was divided by the total incident photon energy to calculate the tPBM penetration rate.

### Behavioral tests

#### Novel object recognition test

The NOR test, based on rodents’ inherent preference for novelty, was utilized to evaluate cognitive function, specifically recognition and memory [54,55]. The procedure was conducted in a 40 cm × 40 cm × 50 cm open-field box (Fig. 1C). Following a habituation session where mice were placed in the test room, 2 identical nontoxic cylinder objects were introduced into the arena 24 h later. Each mouse explored these familiar objects for 10 min, with movements captured by an overhead camera. After a 1-h intertrial interval, one familiar object was replaced by a novel cuboid of identical height and volume, and mice were allowed another 10-min exploration. Object exploration time, trajectory, and total distance traveled were continuously recorded using Tracking Master-V4.0-YM (Shanghai Fanbi Intelligent Technology Co., Shanghai, China). Cognitive performance was quantified using the discrimination index (DI = (*T*_novel_ – *T*_familiar_)/(*T*_novel_ + *T*_familiar_), where *T*_novel_ and *T*_familiar_ denote exploration time for the novel and familiar objects, respectively). Average velocity and total distance traveled were also analyzed to account for potential locomotor differences.

#### Morris water maze test

The MWM test, a widely adopted paradigm for assessing spatial learning and memory [56,57], was conducted in a 1.5-m-diameter circular pool. The pool was filled with 30 cm of water, maintained at 25 ± 1 °C, and rendered opaque with white edible pigment to obscure a hidden escape platform positioned 2 to 3 cm below the surface (Fig. 1D). The pool was conceptually divided into 4 quadrants. All mice underwent an acquisition phase consisting of 4 daily trials for 5 consecutive days. In each trial, mice were randomly introduced into a different quadrant and allowed up to 60 s to locate the hidden platform. Upon successfully finding the platform or after the 60-s trial expired, mice were allowed to remain on the platform for 15 s to consolidate spatial cues. Escape latency (time to reach the platform) and swimming speed were recorded during this acquisition phase. Twenty-four hours after the final acquisition trial, a 60-s probe trial was performed with the hidden platform removed from the tank. Spatial memory was assessed by measuring the percentage of time spent in the target quadrant (where the platform was previously located) and the number of times the mouse crossed the former platform location. Throughout all experimental phases, mouse swimming paths were continuously tracked by an overhead camera system (Tracking Master-V4.0-MWM; Shanghai Fanbi Intelligent Technology Co.) positioned approximately 2.5 m vertically above the center of the pool.

### Brain-tissue preparation

Upon completion of behavioral tests, mice were deeply anesthetized and transcardially perfused with ice-cold phosphate-buffered saline (PBS) to clear blood, followed by fixation with 4% paraformaldehyde (PFA). Brains were then carefully extracted. For immunohistochemical analysis, one hemisphere was immersion fixed in 4% PFA at 4 °C for 24 to 48 h. For biochemical assays, the contralateral hemisphere was rapidly dissected into specific brain regions (e.g., hippocampus and cortex), snap frozen in liquid nitrogen, and stored at −80 °C until further processing.

### Enzyme-linked immunosorbent assay

For Aβ quantification, brain tissue from the snap-frozen hemispheres was utilized. The mouse amyloid beta peptide 1-42 (Aβ_1-42_) levels were assessed using an enzyme-linked immunosorbent assay kit (No. CSB-E10787m; CUSABIO) according to the manufacturer’s instructions. Briefly, approximately 50 mg of brain tissue was homogenized in 450 μl of PBS. Following thorough grinding and centrifugation, the resulting supernatants were collected, and their absorbance was measured at 450 nm using a microplate reader.

### Immunofluorescence

The paraffin-embedded mouse brain was sectioned coronally at 4 μm thickness using a pathology microtome. Sections were then deparaffinized by sequential immersion in xylene (twice, 20 min each), followed by graded ethanol washes (anhydrous ethanol twice, 85% ethanol once, and 75% ethanol once, 5 min each), and finally rinsed with distilled water. For antigen retrieval, slides were incubated in citrate antigen retrieval solution (pH 6.0) and heated in a microwave oven (medium-high for 5 min, followed by high heat for 5 min). After cooling to room temperature, sections were washed 3 times (5 min each) in PBS (pH 7.4) with gentle shaking. Subsequently, nonspecific binding was blocked by incubating sections with 5% goat serum (diluted in PBS) for 30 min at room temperature, ensuring even coverage of the tissue.

The primary antibodies were anti-β-Amyloid (D54D2) (1:500, 8243T; Cell Signaling Technology), anti-NeuN (1:200, 66836-1-IG; Proteintech), anti-CD31 (1:2,000, AB182981; Abcam), anti-glial fibrillary acidic protein (GFAP; 1:500, 16825-1-AP; Proteintech), anti-S100B (1:500, OB-PPGP015-02; Oasis Biofarm), anti-neuronal nitric oxide synthase (nNOS; 1:500, OB-PGP070-02; Oasis Biofarm), anti-synaptophysin (1:500, OB-PRB115-02; Oasis Biofarm), anti-Iba1 (1:500, OB-PGP049-02; Oasis Biofarm), and Lycopersicon Esculentum (Tomato) Lectin, DyLight 594 (1:200, DL-1177-1; Vector Laboratories). Sections were immersed in PBS with shaking and washing for 3 times, 5 min each. Thereafter, sections were incubated with goat anti-rabbit IgG H&L (1:1,000, ab6717; Abcam), goat anti-guinea pig IgG, AF488 (1:1,000, G-GP488; Oasis Biofarm), and GoraLite Plus 594-goat anti-rabbit IgG H&L (1:1,000, RGAR004; Proteintech). The sections were soaked in PBS with 0.1 mg/ml DAPI 3 times, 5 min each, with shaking and washing. The sections were sealed with an antifluorescence quenching sealer and imaged by confocal laser scanning microscopy (AX/AX R with NSPARC; Nikon) and a panoramic scanner (Panoramic Scan; 3DHISTECH) with a 20× objective. Images were analyzed using ImageJ software (National Institutes of Health, USA) and Imaris 10.1.0 (Bitplane, Zurich, Switzerland). Co-localization between glial and vascular markers was quantified using Pearson co-localization coefficient in ImageJ. The hippocampus region was differentiated into distinct functional regions, including dentate gyrus (DG), Cornu Ammonis field 1 (CA1), Cornu Ammonis field 2 (CA2), and Cornu Ammonis field 3 (CA3). Given the fuzzy boundary between CA2 and CA3, we combined these 2 regions into a single statistical module (CA23). The regions of interest for statistical analyses were randomly selected based on the DAPI image.

### Fluorescence micro-optical sectioning tomography

After perfusing with cold PBS and PFA, the mouse hearts were then perfused with a mixture of 9 mg fluorescein 5-isothiocyanate (ND420177; Bomeibio), 3 g gelatin, and 30 ml 0.01 M PBS. Before the mouse brains were excised, the mouse carcasses were immersed in cold water for 30 min. The LR White resin (AGR1280A; Agar Scientific) was employed to embed the mouse brain. The samples were rinsed with a graded ethanol series (50%, 75%, 100%, and 100% treatments, each incubated for 2 h) and finally in 100% ethanol for 12 h. The next day, the samples were infiltrated with a graded LR White series (50%, 75%, and 100% treatments, each for 2 h), then in 100% LR White for 48 h. Gelatin capsules were used as an embedding mold to polymerize the tissue block. The LR White was allowed to polymerize at 48 °C for 24 h. The fluorescence micro-optical sectioning tomography (fMOST) system was used to image the mouse brain layer by layer, with a resolution of 0.35 μm × 0.35 μm × 2 μm. The BioMapping 7000 system (Wuhan OE-Bio Co., Ltd.) consisted of a 40× Olympus microscope objective, 488-nm and 561-nm lasers, and a time-delay integration CCD camera, which was used for detecting images from the fMOST system.

We employed Imaris 10.1.0 for whole-brain visualization and local vascular network reconstruction. Downsampled 2-dimensional TIF sequences were converted to .ims format for whole-brain rendering. For detailed analysis, voxel blocks (1,024 × 1,024 × 1,024 pixels) at original resolution were extracted from 3 hippocampal subregions and the cortex. After preprocessing with background removal and Gaussian filtering, the “Filament” module was applied for automated 3-dimensional reconstruction, followed by manual refinement to remove spurious branches and correct centerlines. Finally, quantitative analysis was performed to extract vascular parameters, including vessel diameter, total length, and others [6].

### Statistical analysis

All statistical analyses were performed using GraphPad Prism 9.5 for Windows software. Quantitative results were expressed as mean ± standard error of the mean. One-way or 2-way analyses of variance with Tukey post hoc tests were applied to perform statistical comparisons between groups of behavioral tests and immunofluorescence. Significance levels were set at *P* <0.05 for all analyses.

## Results

A tPBM device was designed to deliver NIR light to WT and 5xFAD mice (Fig. 1B). We employed an 810-nm LED as the light source, which is consistent with most tPBM studies [43,44,58]. Visualization of photon distribution further demonstrated that 810-nm light effectively penetrated the mouse brain, with an estimated ~30% of the incident energy reaching the brain parenchyma (Fig. 1E). The cognitive-related brain regions, including the hippocampus and cortex, were within the effective photon penetration range and considered primary functional targets of tPBM. Two intervention groups were established to systematically compare the therapeutic effects of 2 photon delivery patterns, including CW and 40-Hz light (50% duty cycle). To evaluate potential confounding thermal effects, we monitored scalp temperature changes during tPBM. We found that the temperature did not significantly increase, and no visible erythema or tissue damage was observed.

### NIR light improved cognitive deficits in 5xFAD mice

To evaluate the effect of NIR light on cognitive function in 5xFAD mice, we conducted behavioral assessments after 1-month tPBM (Fig. 1A). The NOR test showed comparable average velocity and total distance across all experimental groups (Figs. S1A and B). The DI was calculated to assess short-term memory and learning ability, but no statistically significant differences in DI were observed between the groups (Fig. 1G). Notably, qualitative analysis of locomotion trajectories (Fig. 1F) revealed that 5xFAD mice in the CW and 40-Hz groups showed a stronger tendency to explore the novel object.

The long-term spatial memory and learning ability were further assessed via a 6-d MWM test (Fig. 1D). During the acquisition phase, both CW and 40-Hz groups exhibited improved memory performance compared to 5xFAD + sham mice. Specifically, on day 3, both CW and the 40-Hz group showed significantly shorter escape latencies compared to 5xFAD controls (5xFAD + sham vs. 5xFAD + CW: 57.98 ± 1.67 s vs. 48.32 ± 3.69 s, *P* = 0.024; 5xFAD + sham vs. 5xFAD + 40 Hz: 57.98 ± 1.67 s vs. 45.92 ± 0.80 s, *P* = 0.007; Fig. 1I). On day 4, the swimming paths of CW and 40-Hz groups were both comparable to that of WT + sham mice (Fig. 1H), but only the 40-Hz group maintained reduced escape latencies compared to 5xFAD + sham mice (5xFAD + sham vs. 5xFAD + 40 Hz: 52.09 ± 2.15 s vs. 39.45 ± 4.05 s, *P* = 0.049; Fig. 1I). Although escape latency generally decreased across training days for all groups, no significant intergroup differences were observed in the proportion of residence time in the target quadrant (where the platform was located) or average swimming speed during the acquisition phase (Fig. 1J and Figs. S1C and D). Furthermore, MWM indicators on the test day (removed platform) showed no significant differences among groups (Figs. S1E to H). Overall, these behavioral results indicated that both CW and 40-Hz light significantly enhanced spatial memory acquisition in 5xFAD mice. However, 40-Hz light appeared to elicit a numerically greater improvement, though this difference was not statistically significant.

### NIR light reduced Aβ deposition in 5xFAD mice

**Fig. 2.**
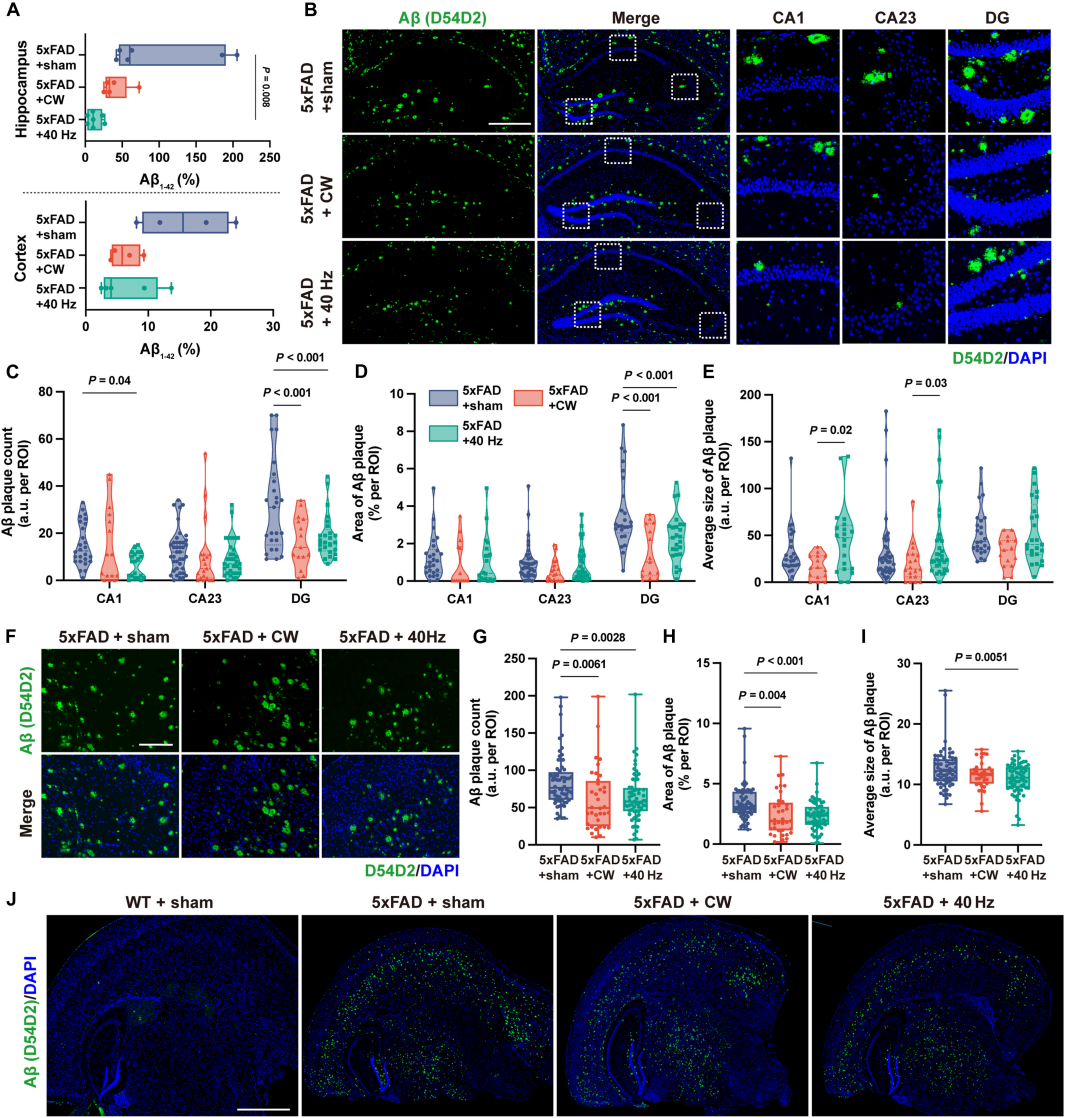
Evaluation of amyloid-β (Aβ) deposition in 5xFAD mice subject to CW and 40-Hz light. (A) Relative Aβ_1-42_ levels in the hippocampus and cortex. (B) Representative images of brain sections in hippocampus stained with anti-Aβ (D54D2, green) and DAPI (blue) in 5xFAD mice. Scale bar = 500 μm. (C to E) The count (C), area ratio (D), and average size (E) of Aβ in each hippocampus subregion, including CA1, CA23, and DG. ROI, region of interest. (F) Representative images of brain sections in the cortex stained with anti-Aβ (D54D2, green) and DAPI (blue) in 5xFAD mice. Scale bar = 200 μm. (G to I) The count (G), area ratio (H), and average size (I) of Aβ in the cortex. (J) Aβ deposition in the coronal section of the hemibrain. Scale bar = 1,000 μm. Data in (A), (C) to (E), and (G) to (I) are analyzed by 1-way or 2-way ANOVA with Tukey post hoc tests of multiple groups.

To explore the effects of NIR light on amyloid pathology in 5xFAD mice, we detected Aβ_1-42_ levels in the cortex and hippocampus via enzyme-linked immunosorbent assay (Fig. 2A). The results showed that 40-Hz light significantly decreased hippocampal Aβ_1-42_ level (87.66%), while CW light exhibited only a mild Aβ_1-42_ clearance effect (60.11%) in the hippocampus. In the cortex, neither stimulation modality produced a statistically significant reduction in Aβ_1-42_ levels, but a downward trend was observed in both CW and 40-Hz groups relative to the 5xFAD + sham mice.

To further investigate Aβ burden of specific brain regions, we employed immunofluorescence with Aβ-specific antibody to semiquantitatively assess Aβ deposition in the hippocampus and cortex. We observed that Aβ plaques were predominantly deposited in the DG region relative to CA1 and CA23 (Fig. 2B). Unexpectedly, among the 3 hippocampal subregions, the DG showed the most robust response to both stimulation protocols, exhibiting significant reductions in Aβ plaque number (CW: 47.56%, 40 Hz: 41.12%; Fig. 2C) and Aβ area proportion (CW: 59.69%, 40 Hz: 37.52%; Fig. 2D). Moreover, 40-Hz light significantly reduced the number of Aβ plaques in CA1 (55.99%, Fig. 2C), while CW light did not significantly reduce Aβ burden in this region. CA23 demonstrated the least response to the therapeutic intervention of both CW and 40-Hz light, showing no significant differences across most indicators (Figs. 2C to E). Notably, compared with 40-Hz light, CW irradiation led to a lower average Aβ plaque size in CA1 and CA23 (Fig. 2E). In the cortex (Figs. 2F and J), although the Aβ plaque load did not return to WT baseline levels, both CW and 40-Hz irradiation significantly decreased the number of Aβ plaques compared to the 5xFAD + sham group (CW: 27.28%, 40 Hz: 24.94%; Fig. 2G). The average size of Aβ plaques and the total Aβ plaque area in the cortex were also remarkably reduced in the 40-Hz group compared to the 5xFAD + sham group (average size: 12.40%, total area proportion: 32.15%; Figs. 2H and I). In contrast, the CW group exhibited a significantly lower total plaque area, but the average plaque size remained comparable to that of the 5xFAD + sham group (average size: 7.55%, total area proportion: 28.80%).

### NIR light improved vascular impairment in 5xFAD mice

**Fig. 3.**
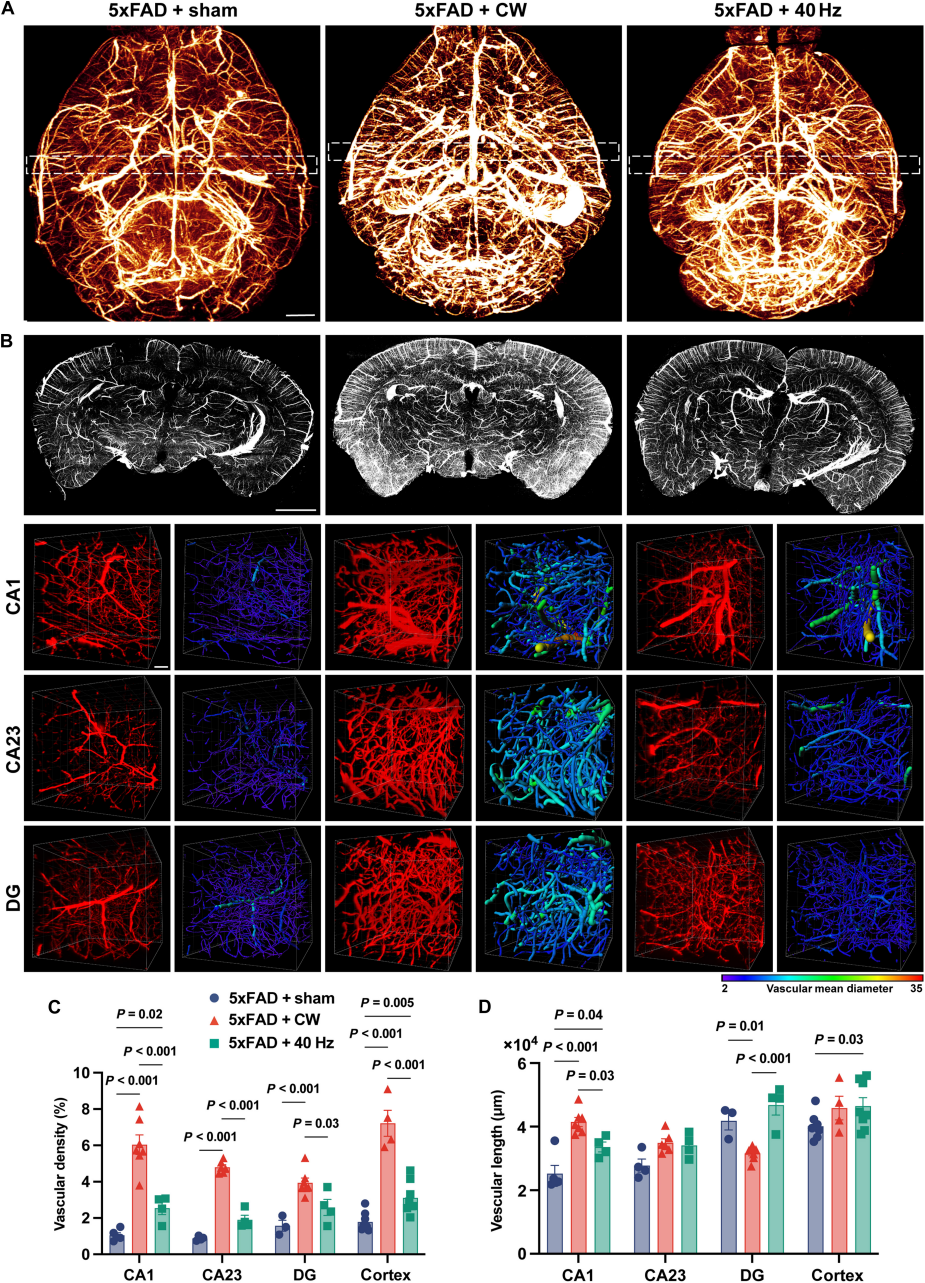
Effect of CW and 40-Hz light on the vascular network in 5xFAD mice. (A) Whole-brain vascular network visualized by fluorescence micro-optical sectioning tomography imaging. Scale bar = 1,000 μm. (B) Representative 3-dimensional reconstruction images of the vascular network in 3 subregions of the hippocampus, including CA1, CA23, and DG. The pseudo-color in reconstructed images represents the vascular mean diameter. Scale bar = 500 μm. (C and D) The vascular density (C) and total vascular length (D) in the hippocampus and cortex. Data in (C) and (D) are analyzed by 2-way ANOVA with Tukey post hoc tests of multiple groups.

To assess whether NIR light improved the cognitive function in 5xFAD mice via mitigating vascular dysfunction, we marked and reconstructed the vascular network of the whole brain (Fig. 3A). Qualitative analysis revealed that compared to the control group, both CW and 40-Hz light increased the density of small blood vessels while having a minimal effect on large vessels. Moreover, the local vessel images were extracted and reconstructed to analyze the vessel density, vessel length, and vessel mean diameter in different brain regions, including CA1, CA23, DG, and cortex (Fig. 3B and Fig. S2A). Specifically, vessel density (the ratio of vascular to total volume) served as a proxy for blood flow and oxygen supply [6,59], vessel length (sum of segment lengths) reflected vascular network complexity, and vessel mean diameter (average segment thickness) functioned as an indirect indicator of perfusion area.

The quantitative results based on fMOST revealed that NIR light significantly improved vascular impairment of the hippocampus and cortex in 5xFAD mice. Compared to the control group, both CW and 40-Hz groups exhibited significantly higher vessel density (Fig. 3C) and increased vessel mean diameter (Fig. S2B). In contrast, vascular length exhibited significant regional heterogeneity, potentially due to the inclusion of major vessels within the regions of interest (Fig. 3D). Specifically, 40-Hz light significantly increased the vascular length in CA1 (33.19%) and cortex (15.77%), while showing a modest enhancement in CA23 (22.84%) and DG (11.83%). Conversely, while CW light increased vascular length in CA1 (64.44%), it significantly reduced vascular length in DG (−24.44%). We also found that both CW and 40-Hz light decreased vascular straightness in CA1 (CW: −2.37%, 40 Hz: −1.60%; Fig. S2C).

### NIR light promoted glial cell response in 5xFAD mice

**Fig. 4.**
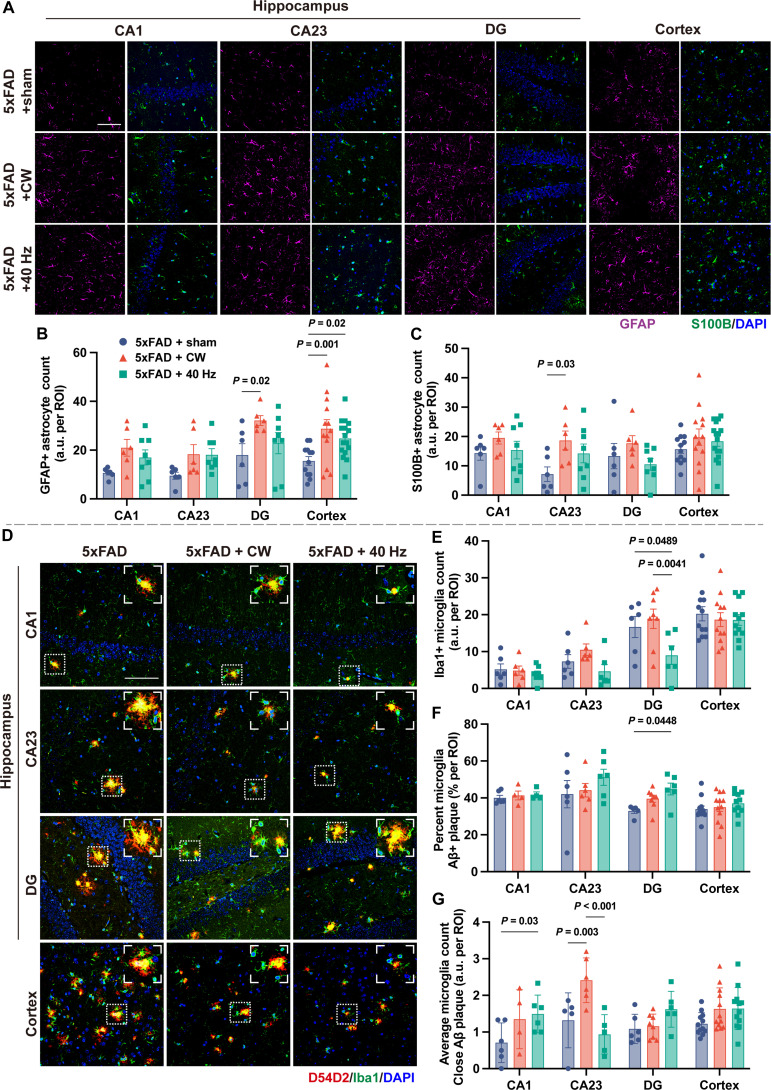
Glial cell response for CW and 40-Hz light in 5xFAD mice. (A) Immunofluorescence with anti-glial fibrillary acidic protein (anti-GFAP, purple) and anti-S100B (green) antibodies in the hippocampus and cortex of 5xFAD mice. (B and C) Number of GFAP+ (B) and S100B+ (C) astrocytes per ROI in the hippocampus and cortex. (D) Immunofluorescence with anti-Aβ (D54D2, red) and anti-Iba1 (green) antibodies in the hippocampus and cortex of 5xFAD mice. (E) Number of Iba+ microglia per ROI in the hippocampus and cortex. (F) Percentage of Aβ amyloid that are also Iba1+ microglia in the hippocampus and cortex. (G) Average number of Iba+ microglia close to Aβ amyloid per ROI in the hippocampus and cortex. Data in (B), (C), and (E) to (G) are analyzed by 2-way ANOVA with Tukey post hoc tests of multiple groups. Scale bar = 100 μm.

Glial cells are sensitive to respond to light stimulation and play a vital role in mediating its biological effects [23,24,44]. To further investigate the effects of CW and 40-Hz light on glial cells, we performed immunostaining for the astrocyte markers (GFAP and S100B) and microglia marker (Iba1) in the hippocampus and cortex (Figs. 4A and D). We observed that both CW and 40-Hz light significantly increased the number of GFAP+ astrocytes (CW: 84.62%, 40 Hz: 58.82%; Fig. 4B) and their area fraction (CW: 73.05%, 40 Hz: 58.95%; Fig. S3A) in the cortex. Beyond this general increase, CW-light irradiation specifically enhanced astrocyte populations in hippocampal subregions, increasing GFAP+ cell number by 78.70% and area fraction by 90.35% in the DG (Fig. 4B and Fig. S3A). Furthermore, GFAP+ astrocyte size in the CA23 region showed a 33.60% increase with CW light (Fig. S3B). S100B+ astrocytes exhibited even more pronounced expansion in CA23, with cell number and area fraction expanding by 160.47% and 245.53%, respectively (Fig. 4C and Fig. S3C). However, neither tPBM modality induced significant effects on the average size of S100B+ astrocytes across all brain regions examined (Fig. S3D).

Regarding microglial responses, quantitative analysis of Iba1+ microglia revealed that 40-Hz light selectively reduced the total number of microglia in the DG subregion by 46.00% (Fig. 4E), whereas CW light showed no significant effects on microglial morphology (Figs. S3E and F) or number. Given the established role of microglia in interacting with Aβ plaques, we further assessed their spatial association with plaques by quantifying the area fraction of microglia within plaques and the average number of microglia surrounding them. Intriguingly, despite the reduction in total cell numbers, 40-Hz light exhibited a higher microglial density tightly surrounding Aβ plaques in the DG compared to the 5xFAD + sham group (Fig. 4F). In the hippocampal CA1 region, 40-Hz stimulation doubled the number of peri-plaque microglia (2-fold increase, *P* = 0.03; Fig. 4G), while CW irradiation induced a 2.5-fold elevation in microglial clustering around plaques in CA23 (*P* = 0.003). These results suggested that tPBM with NIR light robustly activated both astrocyte and microglia, consistent with prior light stimulation studies [23,24]. However, we further revealed that tPBM with CW and 40-Hz light exhibited complementary glial cell selectivity: CW irradiation predominantly drove astrocytic activation, whereas 40-Hz light specifically potentiated microglia-mediated Aβ clearance.

### NIR light induced connections between glial cells and cerebrovascular in 5xFAD mice

**Fig. 5.**
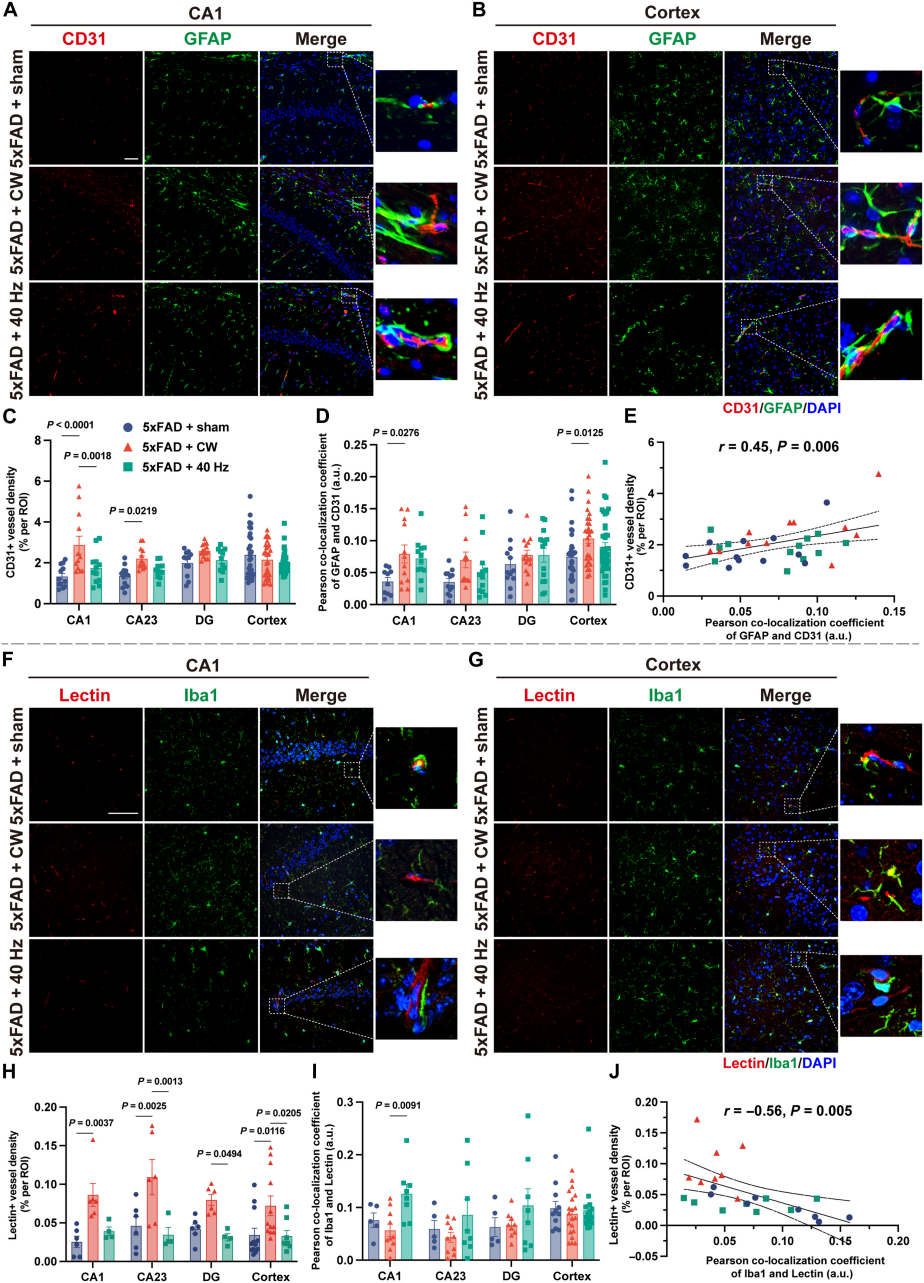
Assessment of gliovascular interactions in 5xFAD mice subject to CW and 40-Hz light. (A and B) Immunofluorescence with anti-CD31 (red) and anti-GFAP (green) antibodies in the CA1 (A) and cortex (B) of 5xFAD mice. (C) CD31+ vessel density in the hippocampus and cortex. (D) Pearson co-localization coefficient of GFAP and CD31 in the hippocampus and cortex. (E) The Pearson correlation analysis between CD31+ vessel density and the Pearson co-localization coefficient of GFAP+ astrocytes and CD31+ vessel. (F and G) Immunofluorescence with Lectin (red) and anti-Iba1 (green) antibodies in the CA1 (F) and cortex (G) of 5xFAD mice. (H) Lectin+ vessel density in the hippocampus and cortex. (I) Pearson co-localization coefficient of Iba1 and Lectin in the hippocampus and cortex. (J) The Pearson correlation analysis between Lectin+ vessel density and the Pearson co-localization coefficient of Iba1+ microglia and Lectin+ vessel. Data in (C), (D), (H), and (I) are analyzed by 2-way ANOVA with Tukey post hoc test of multiple groups. Scale bar = 50 μm.

Astrocytes and microglia have been demonstrated to participate in the regulation of the cerebrovascular network [29,30,32,39]. Thus, to determine whether NIR-light-mediated vascular improvements involved glial activation, we conducted co-immunostaining of vascular markers (CD31 or Lectin) and glial markers (GFAP and Iba1). The quantitative co-localization analysis was performed to evaluate the connection between glial cells and vessels. We observed that 2-dimensional CD31 (Fig. 5C) and Lectin (Fig. 5H) labeling confirmed CW-induced improvement in vascular density, consistent with 3-dimensional quantification results from fMOST.

The co-localization analysis revealed that CW light significantly enhanced the spatial coupling between GFAP+ astrocytes and CD31+ vessels. Specifically, a significant increase in GFAP–CD31 Pearson co-localization coefficient was observed in both the CA1 (5xFAD + sham vs. 5xFAD + CW 0.04 ± 0.01 vs. 0.08 ± 0.01, *P* = 0.028; Figs. 5A and D) and cortex regions (5xFAD + sham vs. 5xFAD + CW: 0.07 ± 0.01 vs. 0.10 ± 0.01, *P* = 0.013; Figs. 5B and D), while no significant changes were found in CA23 and DG (Figs. S4A and B). The increased proportion of CD31 co-localized structures (Fig. S4D), occurring without proportional changes in GFAP co-localization (Fig. S4E), suggested that astrocytic increase induced by CW light facilitated more process extension toward vascular endothelia (increased co-localized area of GFAP and CD31; Fig. S4C). Moreover, the positive correlation (*r* = 0.34, *P* = 0.086; Figs. S6A and B) between GFAP+ astrocyte number and the GFAP–CD31 Pearson co-localization coefficient further corroborated this observation. We also found that this Pearson co-localization coefficient of GFAP and CD31 was positively correlated with CD31+ vessel density (*r* = 0.45, *P* = 0.006; Fig. 5E). Together, these data indicated that the vascular protective effects of CW stimulation were likely mediated through enhanced astrocyte–vascular coupling.

In contrast, no significant overall effects of NIR light were observed on the spatial association between microglia and vessels (Figs. 5F and G and Figs. S4F and G) when compared to the 5xFAD + sham group. The Iba1–Lectin co-localization level showed only a slight increase in CA1 region of the 40-Hz group compared to the CW group (Fig. 5I and Figs. S4H to J). However, we also observed that the Pearson co-localization coefficient between Iba1 and Lectin showed a negative correlation with Lectin+ vessel density (*r* = −0.56, *P* = 0.005; Fig. 5J). This is primarily due to NIR-light-induced augmentation of vascular density without a concomitant increase in microglia. Additionally, this Pearson co-localization coefficient also positively correlated with GFAP and CD31 coupling (*r* = 0.45, *P* = 0.015; Fig. S6E), which may involve synergistic effects secondary to increased vascular density.

### NIR light improved synaptic impairment via activating glial cell–vascular coupling in 5xFAD mice

**Fig. 6.**
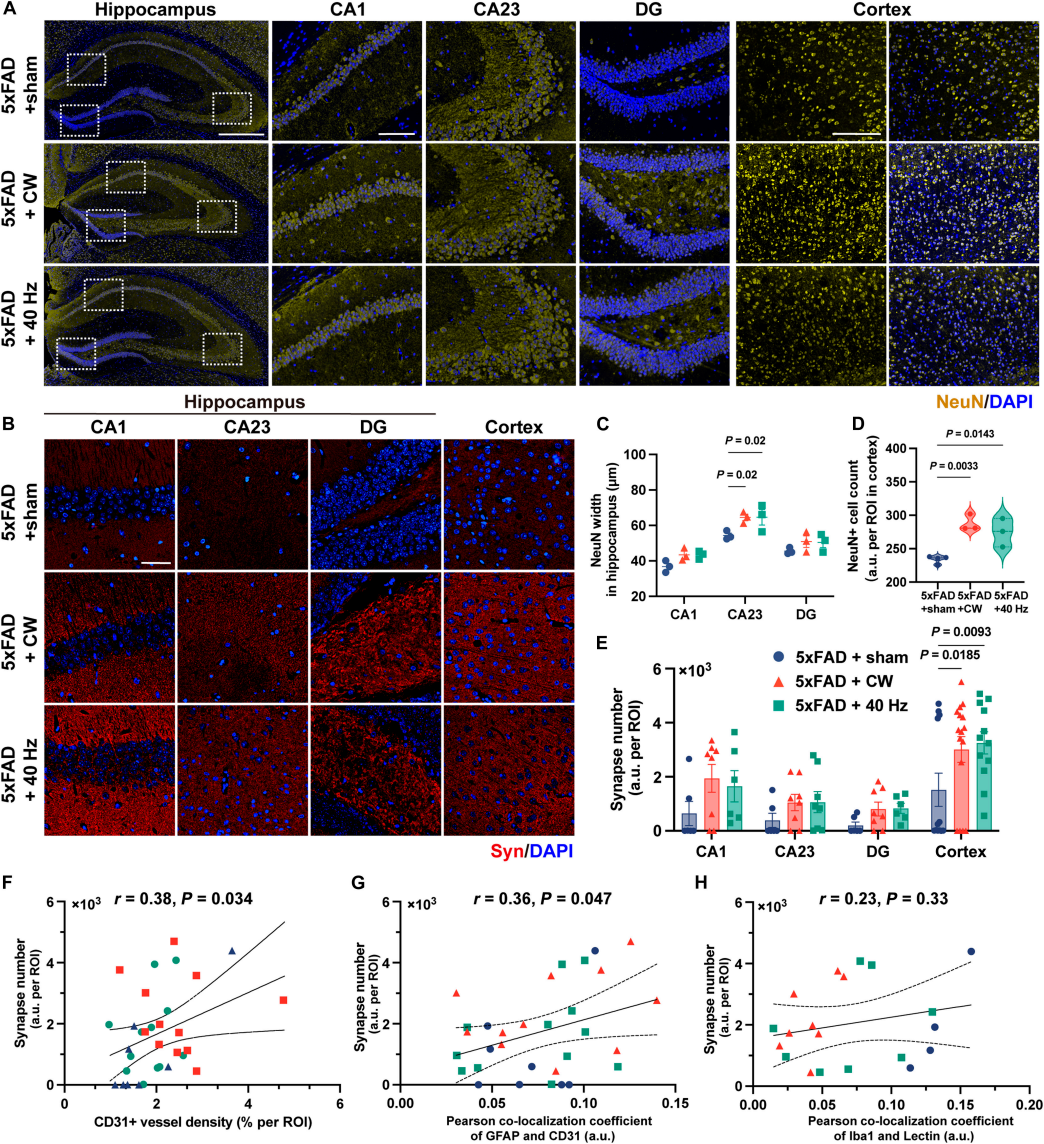
Effect of near-infrared light on neurodegenerative pathology in 5xFAD mice. (A) The representative images were immunofluorescence with anti-NeuN (yellow) and DAPI (blue) in the hippocampus and cortex of 5xFAD mice. (B) The representative images were immunofluorescence with anti-Syn (red) and DAPI (blue) in the hippocampus and cortex of 5xFAD mice. Scale bar = 50 μm. (C) The neuron bandwidth in the hippocampus. (D) The neuron number in the cortex. (E) The synapse number in the hippocampus and cortex. (F) The Pearson correlation analysis between synapse number and vessel density. (G) The Pearson correlation analysis between synapse number and the Pearson co-localization coefficient of GFAP+ astrocytes and CD31+ vessels. (H) The Pearson correlation analysis between synapse number and the Pearson co-localization coefficient of Iba1+ microglia and Lectin+ vessel. Data in (C) to (E) are analyzed by 1-way or 2-way ANOVA with Tukey post hoc test of multiple groups.

To evaluate the neuroprotective effects of NIR light, we performed immunofluorescence analyses targeting NeuN+ neurons and synaptophysin+ (Syn) synapses. Due to dense hippocampal neuronal packing, we measured neuronal band widths (50-μm intervals) as a proxy for cell numbers. We found that both CW and 40-Hz NIR light significantly alleviated NeuN+ cell loss in CA23 (CW: 10.57%, 40 Hz: 20.37%; Figs. 6A and C) and increased cortical neuron density (CW: 22.95%, 40 Hz: 17.35%; Figs. 6A and D), while showing no significant effects in CA1 and DG.

Synaptophysin, as a membrane protein localized on synaptic vesicles, participates in synaptic transmission and maintains synaptic plasticity [43]. We observed that both CW and 40-Hz light stimulation significantly increased the number of synapses in the cortex (CW: 98.17%, 40 Hz: 114.17%; Figs. 6B and E). In contrast, only a modest increase in synaptic density was detected in hippocampal subregions (Fig. 6E and Figs. S5A and B). To further investigate the relationships between synapse and glial/vascular components, we analyzed the correlation between synaptic quantitative metrics and glial-associated indicators (Fig. S6A). The results showed that the synapse number positively correlated with both CD31+ vessel density (*r* = 0.38, *P* = 0.034; Fig. 6F) and GFAP–CD31 Pearson co-localization coefficient (*r* = 0.36, *P* = 0.047; Fig. 6G). This suggested that enhanced astrocyte–vascular coupling not only ameliorated vascular damage but also achieved synaptic protection by improving blood supply. Conversely, synaptic quantitative metrics showed no significant correlation with microglia markers or their vascular Pearson co-localization coefficients (Fig. 6H and Fig. S6A). Thus, we inferred that the protective effects of tPBM on synapses were primarily mediated through astrocytes, with no direct involvement of microglia. Furthermore, although previous studies have shown that light stimulation triggers the release of NO [60,61], no significant changes in anti-neuronal nitric oxide synthase were observed in the CW and 40-Hz groups (Fig. S5C).

## Discussion

**Fig. 7.**
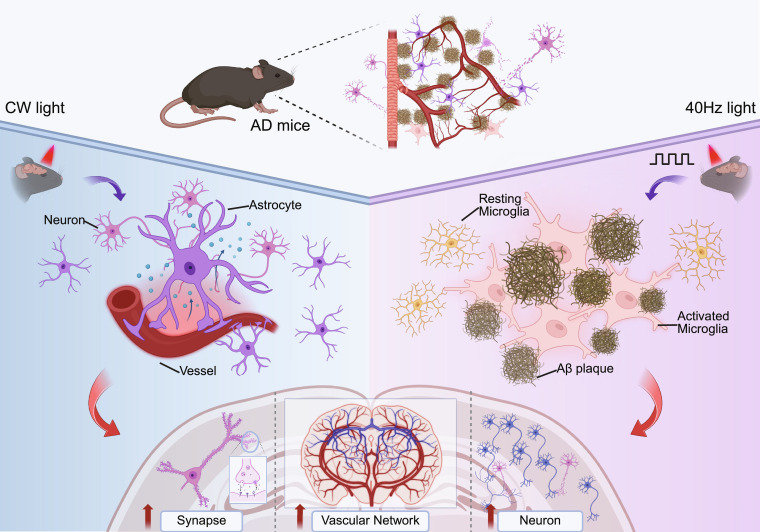
Schematic illustration of frequency-specific tPBM glial mechanisms in AD mice. CW light preferentially enhances astrocyte–vascular coupling to restore vascular and protect synapses, whereas 40-Hz pulsed light promotes microglial redistribution around Aβ plaques to facilitate amyloid clearance and alleviate neurodegenerative pathology.

In this study, we showed that both tPBM modalities improved cognitive deficits, while they engaged distinct glial-mediated modulation pathways (Fig. 7). CW light preferentially enhanced astrocyte–vascular coupling, thereby ameliorating cerebral vasculature impairment and providing indirect synaptic protection. In contrast, 40-Hz light induced spatial redistribution of microglia, driving their targeted migration toward Aβ plaques to enhance local clearance. These results highlight the pathway selectivity of tPBM, suggesting that distinct stimulation modalities can be strategically leveraged to target specific pathological components of AD.

Recent advances in light stimulation for AD have delineated 2 principal paradigms [58], including frequency-entrained neural modulation and mitochondria-targeted tPBM. Iaccarino et al. [23] demonstrated that gamma-frequency visual stimulation reduces AD pathology through neural oscillation entrainment, while Wang et al. [43] found that tPBM with CW light enhanced meningeal lymphatic Aβ clearance via the activation of mitochondrial chromophores. Our work synthesized these 2 modalities, demonstrating that direct transcranial delivery of gamma-frequency light also effectively restores cognitive function in AD mice through eliciting microglial responses.

Notably, the behavioral improvement observed in this study was primarily reflected in the MWM task, which assesses hippocampus-dependent spatial learning and memory. In contrast, no statistically significant differences were detected in the discrimination index of the NOR task. This dissociation suggests that tPBM preferentially enhanced spatial memory rather than broadly improving all cognitive domains under the present experimental conditions. Given that the MWM critically relies on hippocampal circuitry, particularly CA1 function, the improved memory performance aligns with the trend of regional vascular remodeling and enhanced astrocyte–vascular coupling observed in the hippocampus. Protection of vascular network integrity within the hippocampus may improve local perfusion and metabolic support, thereby stabilizing synaptic structure and function, which are essential for spatial memory processing [5,29].

Currently, established tPBM mechanisms mainly focus on photon–chromophore interactions (e.g., cytochrome *c* oxidase activation) [14,62] and a biphasic dose response [63–65]. Thus, within a therapeutic window, treatment efficacy is generally expected to scale with optical dose. However, our findings reveal that 40-Hz light achieved cognitive benefits comparable to those of CW mode, despite delivering only half the total energy (50% duty cycle). This “high-efficiency” profile implies that 40-Hz light likely engages alternative regulatory pathways beyond mitochondrial metabolic effects. Divergent findings in other application scenarios also support this hypothesis. Specifically, CW outperforms pulsed light in wound healing [66], while PW light has shown superior neuroprotection in TBI models [26]. Thus, synthesizing the differential pathways of 2 light modalities revealed in this study, we infer that light stimulation in non-neural tissues primarily relies on photon-driven adenosine triphosphate production, rendering CW light more effective for wound healing. In contrast, PW-based tPBM activates additional biological activities and mechanisms, such as neural entrainment, gamma-frequency triggering of intracellular calcium oscillations, or the gating of specific ion channels in microglia. These mechanisms underlie the therapeutic advantages demonstrated in AD and TBI models, which are likely unattainable with CW light.

Cerebrovascular dysfunction, characterized by diminished perfusion and neurovascular uncoupling, is a critical precipitating factor in AD pathology [29,67,68]. While previous studies explored light-induced angiogenesis [16,69], most relied on 2-dimensional histology, which failed to capture the complexity of the vascular network. By leveraging whole-brain fMOST imaging, we provide a comprehensive 3-dimensional visualization of the cerebrovascular architecture, demonstrating that tPBM induces actual structural remodeling rather than merely transient hemodynamic changes. Notably, we observed that tPBM effectively mitigated the vessel diameter shrinkage typically seen in 5xFAD mice. This structural restoration provides the anatomical substrate for the improved cerebral perfusion reported in hemodynamic studies [70]. Combined with the stable scalp temperature observed during tPBM, these results indicate that the vascular changes induced by tPBM are primarily attributable to its direct photobiomodulatory effects rather than secondary thermal effects caused by temperature. Furthermore, aligning with the regional heterogeneity mapped by Zhang et al. [6], we found that the vascular response to light was not uniform across the brain but varied with baseline vascular complexity. This suggests that tPBM efficacy may be modulated by the underlying angioarchitecture density. Crucially, this vascular recovery, particularly the enhanced vessel diameter, aligns with our observations of astrocyte activation (discussed below). The tPBM-induced vascular improvements are not an isolated phenomenon but a consequence of the enhanced astrocyte–vascular coupling we identified.

Although precise mechanistic pathways through which NIR light modulates vascular architecture and synapses remain incompletely elucidated, existing evidence supported that astrocytes were the anatomical and functional link between vasculature and nervous system [71]. As the central elements of NVU, astrocytes structurally couple the vasculature to neurons, regulating both cerebral blood flow [72] and synaptic transmission [36]. Our findings indicate that CW light preferentially targets astrocytes. Unlike auditory stimulation that robustly activates astrocytes via gamma entrainment [24], CW light likely boosts astrocytic function through mitochondrial metabolic enhancement [44]. This hypothesis is supported by the significant correlation we observed between astrocyte–vascular coupling and synaptic density. Thus, rather than acting directly on synapses, CW light appears to preserve synaptic integrity by reinforcing the NVU structure, thereby ensuring adequate metabolic delivery. In contrast, 40-Hz stimulation exerted its primary effects through microglial modulation, consistent with visual light stimulation outcomes across other studies [22,73,74]. A major challenge in AD therapy is that broad microglial activation often triggers neurotoxic inflammatory cascades [41]. Crucially, our data suggest that 40-Hz light avoids this issue by inducing a targeted microglial state. Instead of global, pro-inflammatory activation, 40-Hz light specifically promoted microglial clustering around Aβ plaques. This localized response allows for efficient plaque clearance while minimizing damage to healthy tissue.

Our findings provided a framework for precision parameter optimization to guide the clinical application of tPBM. The results show that CW light enhances astrocyte–vascular coupling, which may be preferentially indicated for conditions characterized by vascular dysfunction or hypoperfusion, such as vascular dementia [75,76]. The 40-Hz light primarily exerts therapeutic effects through microglial mobilization, making it particularly suitable for early-stage AD where amyloid burden represents an early and therapeutically targetable pathological change. Furthermore, we infer that a combinatorial protocol (alternating or superimposing modes) could theoretically ameliorate both vascular deficits and amyloid burden simultaneously. Future clinical trials are recommended to identify this potential synergistic effect. Beyond parameter optimization, tPBM also offers a practical advantage for translational studies. Electrical or magnetic stimulation can introduce stimulation-related electromagnetic artifacts, particularly during electrophysiological recordings, whereas optical stimulation does not directly introduce electrical currents or magnetic pulses [77,78]. This feature may facilitate the integration of tPBM with multimodal brain monitoring (such as electroencephalography) in future clinical studies.

Furthermore, sex should also be considered when translating these findings, as biological sex can influence AD pathology. In this study, male and female mice were included in a balanced manner across experimental groups, which reduced potential sex-related sampling bias. Previous studies have shown that sex differences can be age and phenotype dependent, with female mice showing higher cortical plaque numbers at several ages and a tendency toward increased microglial density [79]. Moreover, sex-related differences in microglial metabolism and phagocytic function have been reported in human AD tissue [80]. Therefore, while the present results support the overall efficacy of frequency-specific tPBM in this sex-balanced study, future translational studies should further determine whether sex influences the optimal selection of CW, 40 Hz, or combined stimulation protocols.

Some limitations in this study should be acknowledged. First, while we identified astrocytes and microglia as critical cellular mediators for the neurovascular modulation effects of NIR light, these findings are primarily phenotype based. The specific signaling pathways involved warrant further validation through molecular or transcriptomic analysis. Second, although we observed that CW and 40-Hz light preferentially engaged different glial populations to enhance cognition, the mechanistic underpinnings of this selective activation remain unclear. Future studies should investigate the combinatorial effects of CW and PW light, which may enable tPBM strategies to address more diverse pathological features. Region-specific neurobiological effects of tPBM, particularly in cortical subregions such as the prefrontal cortex, should also be further explored to clarify the circuit mechanisms underlying tPBM-mediated cognitive improvement. In addition, electrophysiological and in vitro assays will be required to validate the mechanisms by which light stimulation may engage frequency-dependent cellular responses. Furthermore, determining whether other frequency bands elicit similar or distinct therapeutic effects will be essential for further parameter optimization and clinical translation of tPBM.

In summary, our results revealed that both CW and 40-Hz light comparably enhanced cognitive performance and ameliorated neurovascular pathology, albeit through divergent glial-mediated mechanisms driven by their distinct light delivery patterns. Specifically, CW light predominantly activated astrocytes, which strengthened astrocyte–vascular coupling to improve vascular impairment. In contrast, 40-Hz light primarily orchestrated the spatial redistribution of microglia toward Aβ plaques, thereby significantly enhancing Aβ clearance and protecting synapses. Collectively, our work demonstrated the biological pathway selectivity of specific light delivery modalities in tPBM, which provided a fundamental framework for developing precision tPBM strategies.

## Data Availability

All the data are available upon request on the corresponding author.
