# Supplementary material for: Frequency-Specific Transcranial Photobiomodulation Elicits Complementary Glial Mechanisms for Neurovascular Protection and Amyloid Clearance in Alzheimer Disease
Source: Cyborg Bionic Syst. 2026 Jun 23;7:0551. doi: 10.34133/cbsystems.0551 (PMC13287457; doi:10.34133/cbsystems.0551)
Supplement: Supplementary 1 — Figs. S1 to S6 Table S1 [file cbsystems.0551.f1.docx]

**Frequency-Specific Transcranial Photobiomodulation Elicits Complementary Glial Mechanisms for Neurovascular Protection and Amyloid Clearance in Alzheimer's Disease**

Bowen Zhang ^1,†^, Zemeng Chen ^1,†^, Weiguang Li ^3,†^, Louzhe Xu ^1^, Songqi Yang ^1^, Felix Wang ^6^, Kai Yan ^5,^*, Xunbin Wei ^4,^*, Ting Li ^1,2^*

^1^ Biomedical Engineering Institute, Chinese Academy of Medical Sciences and Peking Union Medical College, Tianjin, 300192, China

^2^ Institute of Intelligent Medicine, Chinese Academy of Medical Sciences & Peking Union Medical College, Beijing, 100730, China.

^3^ Department of Psychology, the State Key Laboratory of Brain and Cognitive Sciences, The University of Hong Kong, Hong Kong SAR 999077, China

^3^ Peking University, China Department of Biomedical Engineering, College of Future Technology, Peking University, Beijing, 100871,

China

Department of Biomedical Engineering, College of Future Technology, Peking University, Beijing, 100871,

China

Department of Biomedical Engineering, College of Future Technology, Peking University, Beijing, 100871,

China

^4^ Department of Biomedical Engineering, College of Future Technology, Peking University, Beijing, 100871, China

^5^ Children Hospital of Fudan University, Shanghai 201102, China

^6^ Department of Biosystems, KU Leuven, Leuven, Belgium

† These authors contributed equally to this work.

* Correspondence to Kai Yan, Xunbin Wei, Ting Li: [liting@bme.cams.cn](mailto:liting@bme.cams.cn) (Tel: +86 180-0212-7296)

# Supplementary information

**Figures**

**Fig. S1** The performance of 5xFAD mice during NOR and MWM.

**Fig. S2** Reconstructed vascular network in prefrontal cortex in 5xFAD mice.

**Fig. S3** Near-infrared light stimulation activated astrocytes and microglia.

**Fig. S4** Near-infrared light stimulation increased the connection of glial cells and vascular network.

**Fig. S5** Near-infrared light stimulation improved he neurodegenerative pathology in 5xFAD mice.

**Fig. S6** The correlation analysis of glial cell activation, vascular network and synapse.

**Tables**

**TableS1** Optical properties of mouse brain tissue type at 810 nm


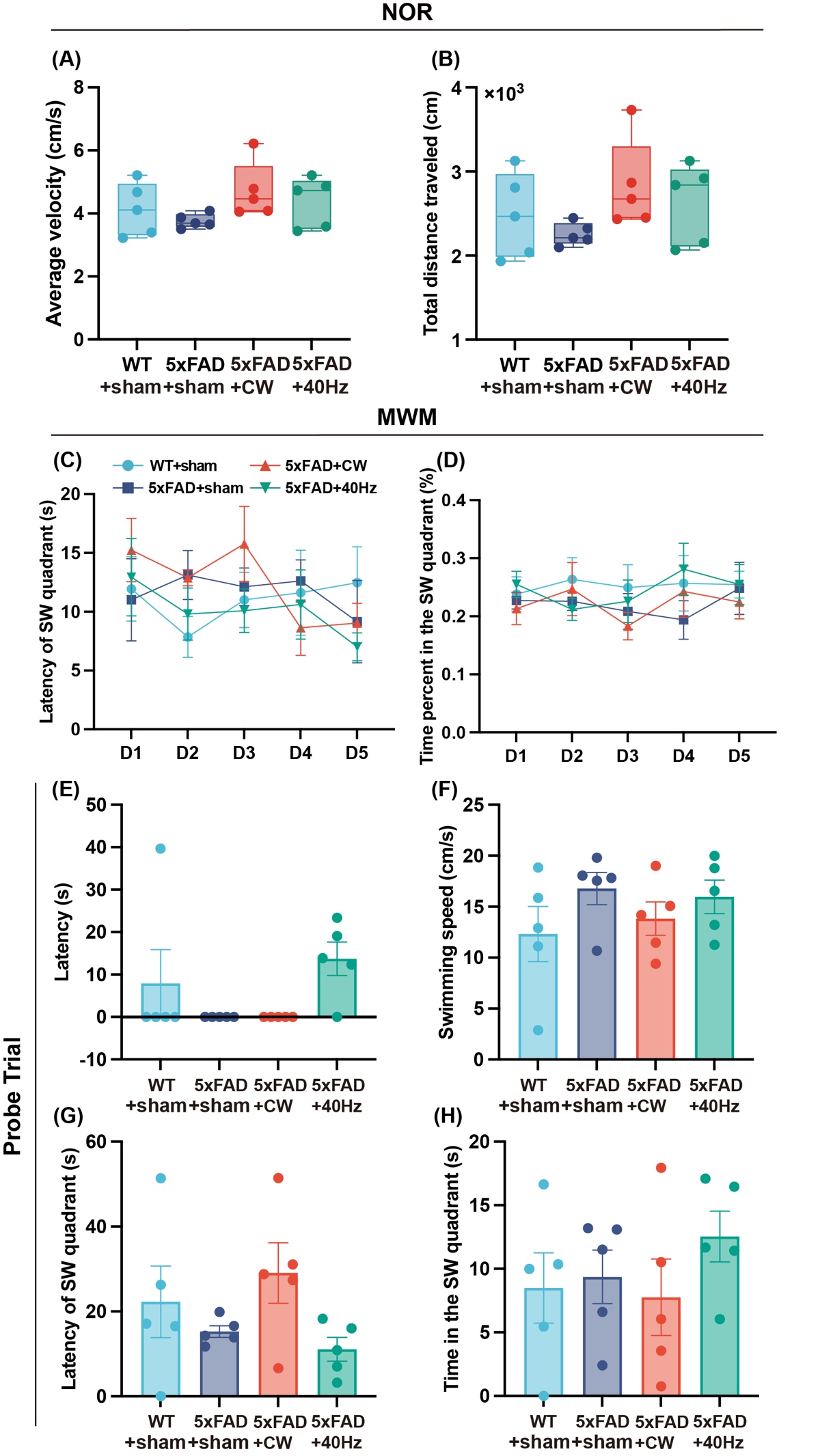


**Fig. S1** **The performance of 5xFAD mice during NOR and MWM.** (A) The average velocity during the NOR test. (B) Total distance traveled during the NOR test. (C, D) The escape latency (C) and the time percent (D) in the southwest (SW) quadrant during 5-day MWM. (E-H) The escape latency (E), swimming speed (F), latency in SW quadrant (G), and swimming time in SW quadrant (H) of mice at the space exploration experiment of Day 6. Data in (A-B, E-H) are presented as mean ± SEM and analyzed by one-way ANOVA with Turkey’s post-hoc tests of multiple groups. Data in (C, D) are analyzed by two-way ANOVA with Tukey’s post-hoc tests of multiple groups.


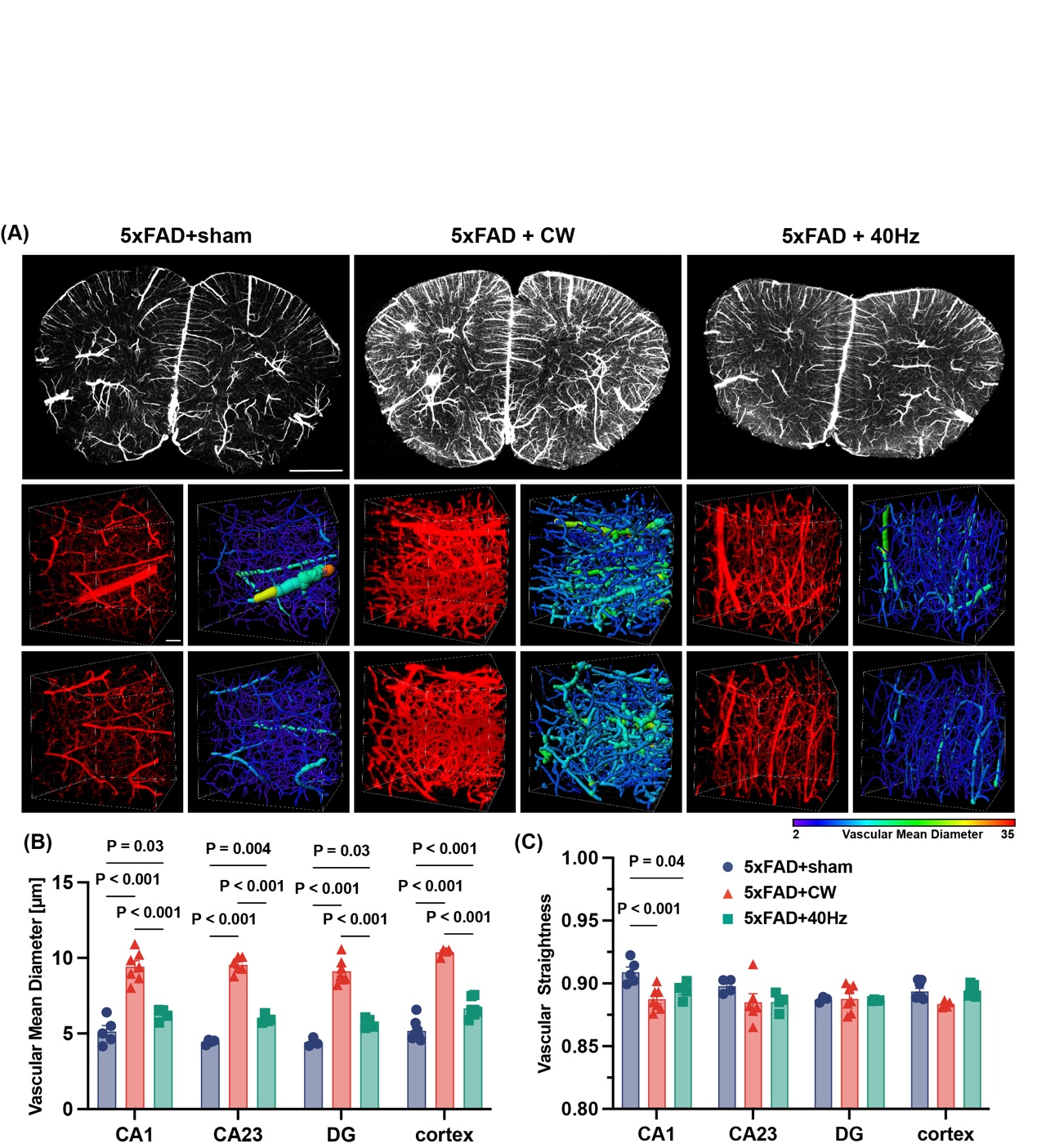


**Fig.S2** **Near-infrared light stimulation improved vascular network impairment in 5xFAD mice.** (A) Representative 3D reconstruction images of vascular network in the prefrontal cortex. The pseudo-color in reconstructed images represents the vascular mean diameter. Scale bar, 1000um. (B, C) The vascular mean diameter (C) and vascular straightness (D) in hippocampus and cortex. Data in (B, C) are presented as mean ± SEM and analyzed by two-way ANOVA with Tukey’s post-hoc tests of multiple groups.


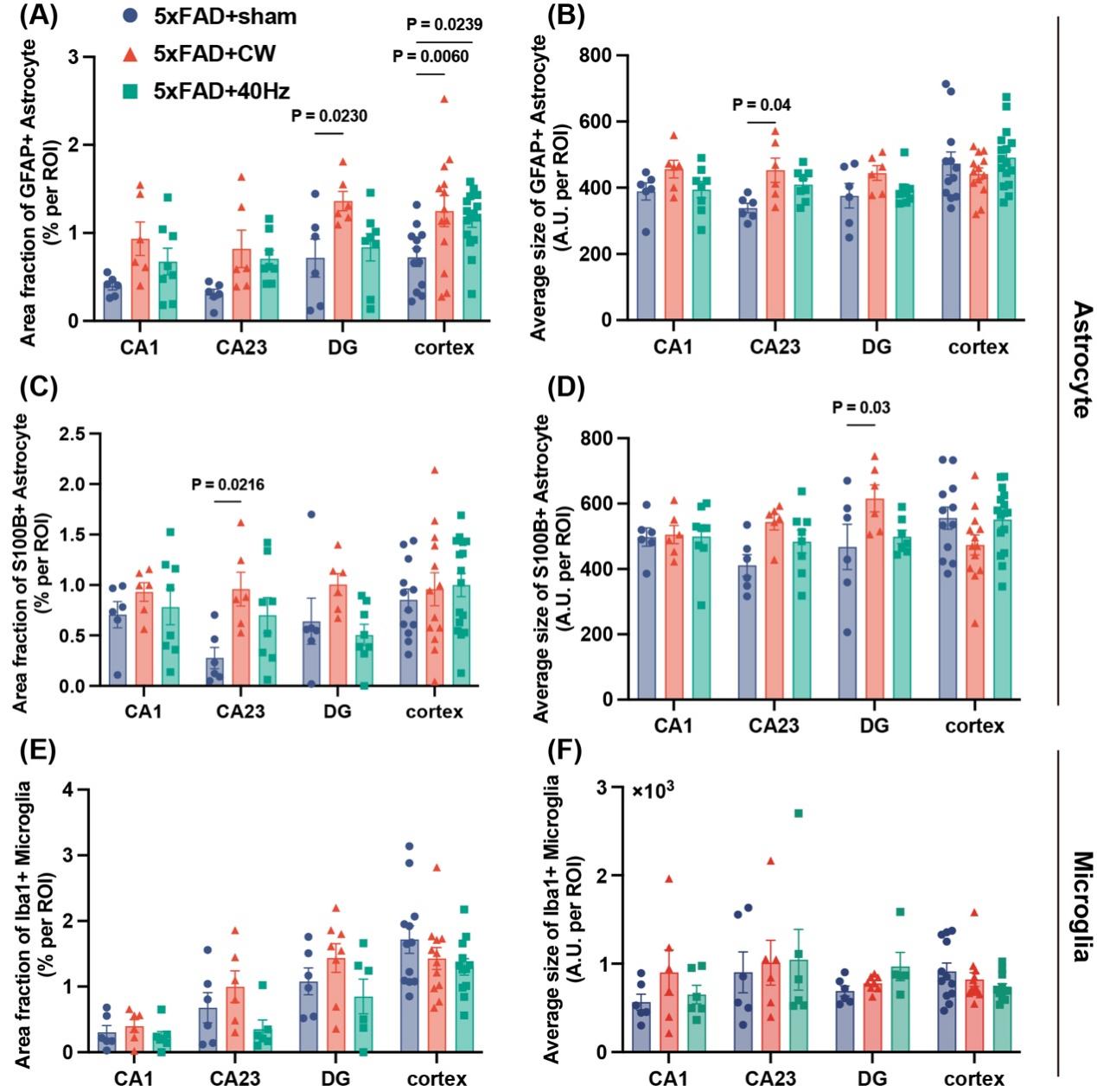


**Fig.S3 Near-infrared light stimulation activated astrocytes and microglia.** (A, B) Area fraction (A) and average size (B) of GFAP+ astrocyte in hippocampus and cortex of 5xFAD mice. (C, D) Area fraction (C) and average size (D) of S100B+ astrocyte in hippocampus and cortex. (E, F) Area fraction (E) and average size (F) of Iba1+ microglia in hippocampus and cortex. Data are presented as mean ± SEM and analyzed by two-way ANOVA with Tukey’s post-hoc tests of multiple groups.


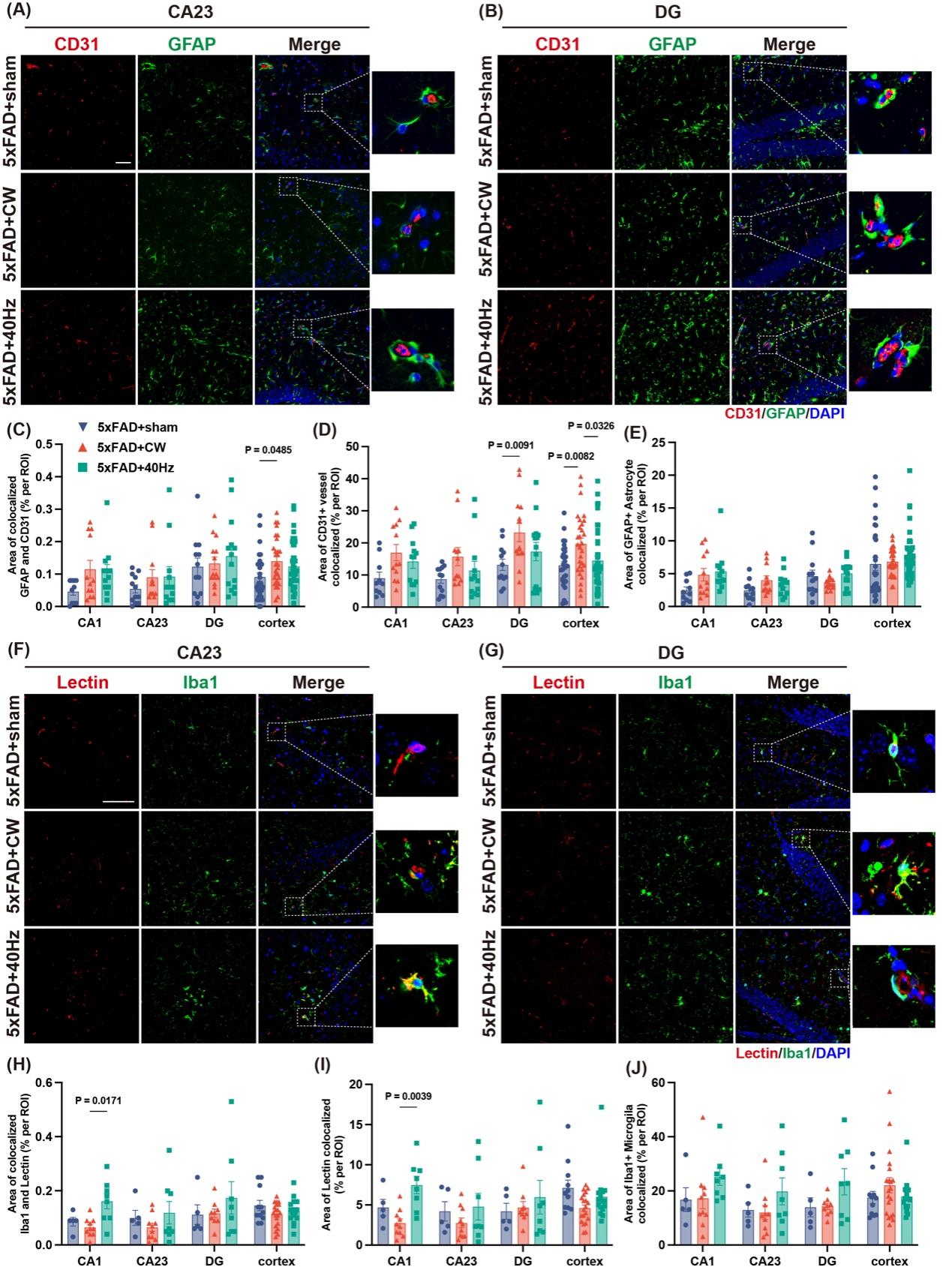


**Fig.S4 Near-infrared light stimulation increased the connection of glial cells and vascular network.** (A, B) Immunofluorescence with anti-CD31 (red) and anti-GFAP (green) antibodies in the CA23 (A) and DG (B) of 5xFAD mice. (C) The area proportion of CD31/GFAP co-localization in hippocampus and cortex. (D) The ratio of CD31 area co-localized with GFAP to total CD31 area. (E) The ratio of GFAP area co-localized with CD31 to total GFAP area. (F, G) Immunofluorescence with Lectin (red) and anti-Iba1 (green) antibodies in the CA23 (F) and DG (G) of 5xFAD mice. (H) The area proportion of Lectin/Iba1 co-localization in hippocampus and cortex. (I) The ratio of Lectin area co-localized with Iba1 to total Lectin area. (J) The ratio of Iba1 area co-localized with Lectin to total Iba1 area. Data in (C-E, H-J) are presented as mean ± SEM and analyzed by two-way ANOVA with Tukey’s post-hoc test of multiple groups.


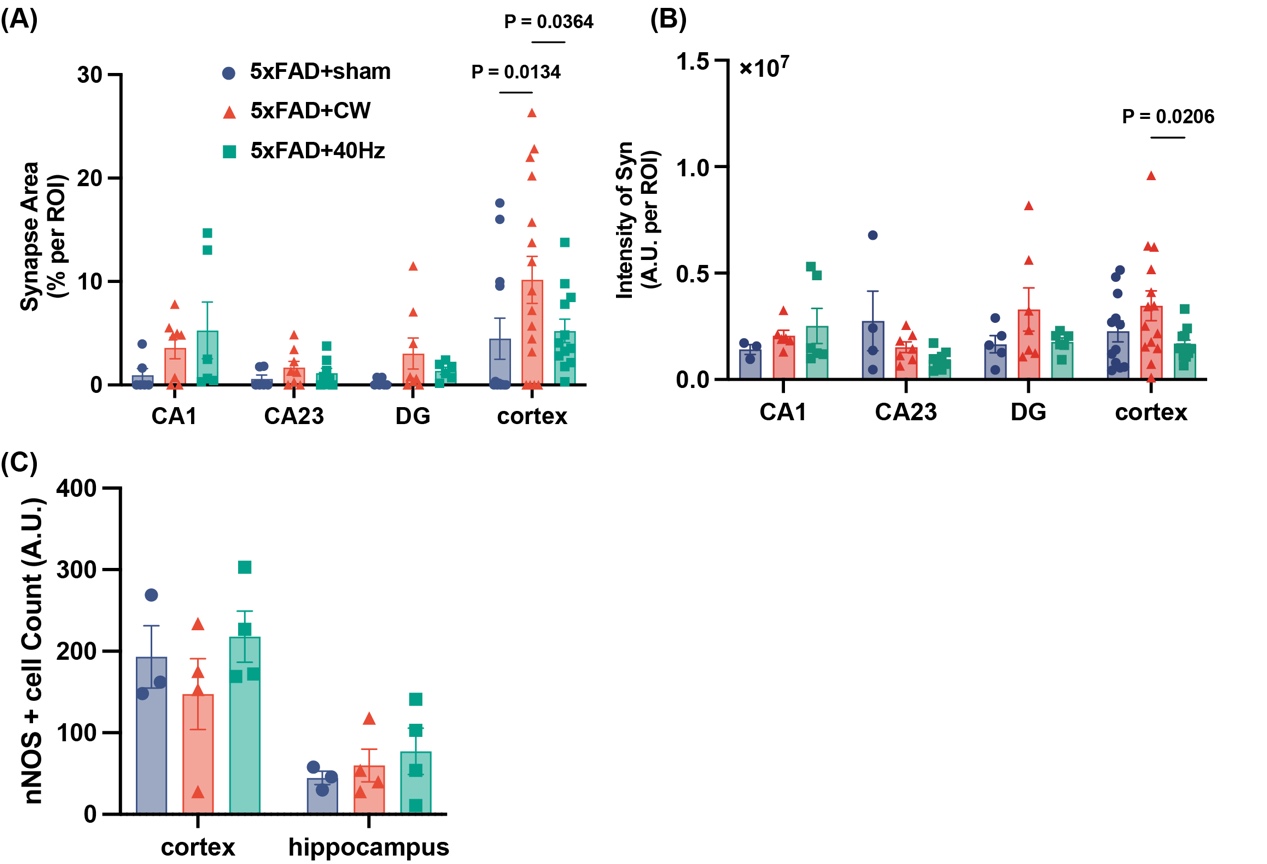


**Fig. S5 Near-infrared light stimulation improved he neurodegenerative pathology in 5xFAD mice.** (A) The synapse area fraction in hippocampus and cortex. (B) The relative intensity of Syn+ signal in hippocampus and cortex. (C) The nNOS+ neuron count in hippocampus and cortex. Data are presented as mean ± SEM and analyzed by two-way ANOVA with Tukey’s post-hoc test of multiple groups.


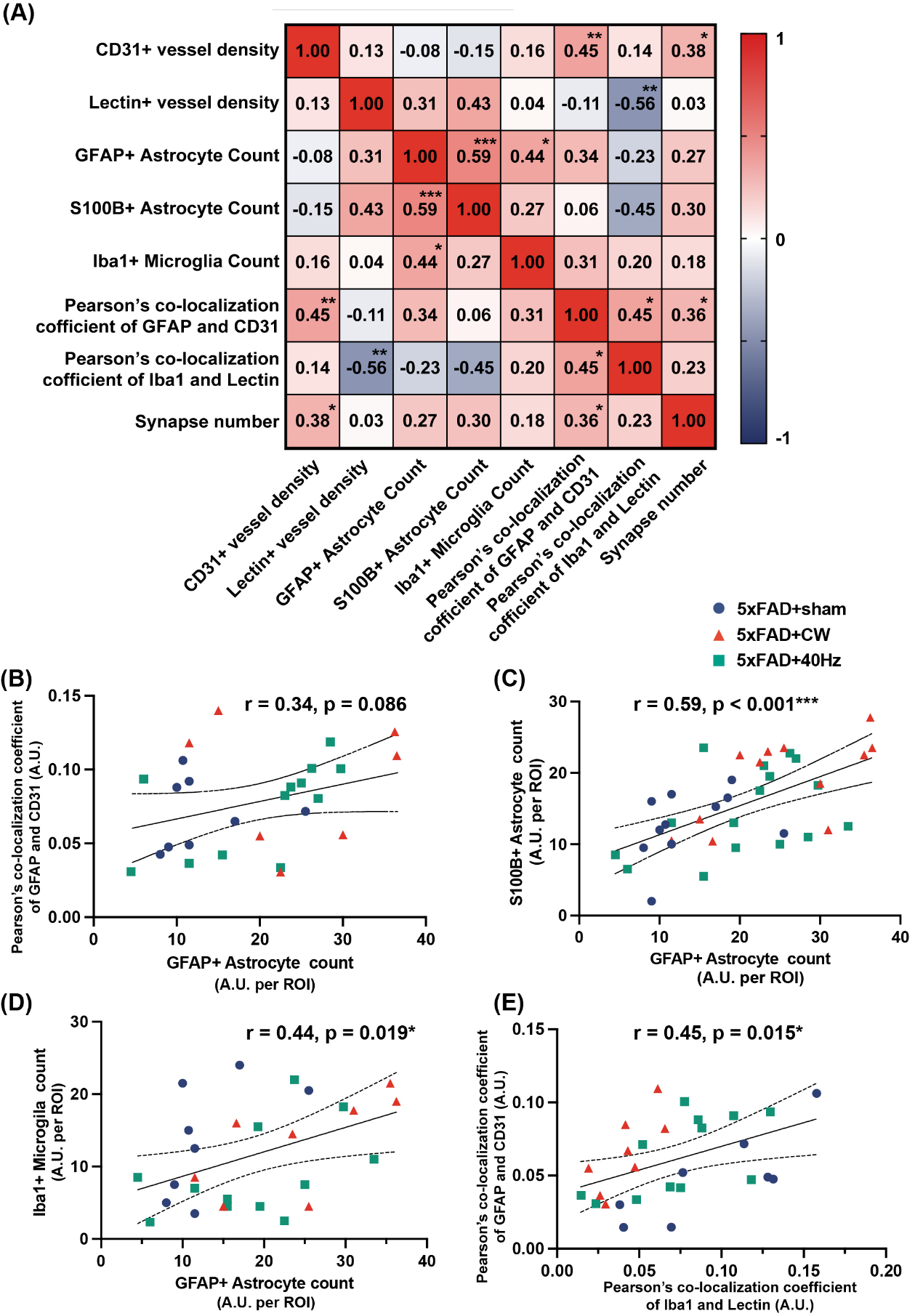


**Fig. S6** **The correlation analysis of glial cell activation, vascular network and synapse.** (A) Correlation matrix among quantitative metrics of glia, vasculature, and synapses. (B) The Pearson’s correlation analysis between GFAP+ astrocyte count and the Pearson’s co-localization coefficient of GFAP+ astrocyte and CD31+ vessel. (C) The Pearson’s correlation analysis between GFAP+ astrocyte count and S100B+ astrocyte count. (D) The Pearson’s correlation analysis between GFAP+ astrocyte count and Iba1+ microglia count. (E) The Pearson’s correlation analysis between the Pearson’s co-localization coefficient of Iba1+ microglia and Lectin+ vessel and the Pearson’s co-localization coefficient of GFAP+ astrocyte and CD31+ vessel.

TableS1 Optical properties of mouse brain tissue type at 810 nm

| Type | n | μ_a_ (cm^-1^) | μ_s_ (cm^-1^) | g |
| --- | --- | --- | --- | --- |
| Scalp | 0.531 | 0.195 | 210 | 0.531 |
| Skull | 1.43 | 0.19 | 173 | 0.91 |
| Muscle | 1.4 | 0.27 | 68.7 | 0.9 |
| Ear | 1.4 | 0.06 | 1.42 | 0.9 |
| Fat | 1.4 | 0.47 | 3.1 | 0.9 |
| CSF | 1.33 | 0.04 | 0.1 | 0.9 |
| Brain parenchyma | 1.37 | 0.28 | 75 | 0.892 |
| Eye | 1.37 | 0.4 | 7 | 0.892 |
